# The Impact of a Lightweight Pneumatic Robotic Knee Wearable on Gait

**DOI:** 10.1155/cro/7160005

**Published:** 2026-02-05

**Authors:** Jonathan J. Lee, Samuel Lyons, Prerna Arora, Jeffery J. Morgan, Salinda Chan, Derek F. Amanatullah

**Affiliations:** ^1^ Department of Orthopaedic Surgery, Stanford University, Redwood City, California, USA, stanford.edu; ^2^ Motion and Sports Performance Laboratory, Lucile Packard Children′s Hospital, Palo Alto, California, USA

**Keywords:** exoskeleton, gait, quadriceps augmentation, robotics, total knee arthroplasty

## Abstract

**Background:**

Exoskeletons may provide quadriceps augmentation to patients with a torn or permanently weak extensor mechanism. Pneumatic exoskeletons utilize pressurized gas to improve gait, but their use and biomechanical impact have not been well described.

**Methods:**

A lightweight pneumatic robotic wearable device was piloted in a clinical gait case report study using four different conditions—no devices, walking stick, inactive brace, and active brace. Walking speed, step length, step time, step width, and hip and knee flexion/extension angles were assessed while walking on a flat, declined, and inclined surface.

**Results:**

Walking speed on a flat surface with the active brace (0.41 ± 0.03 m/s) was 45% faster than walking with no devices (0.28 ± 0.04 m/s). Knee flexion at initial contact (19.0^°^ ± 0.2^°^) and peak knee flexion during stance (19.0^°^ ± 0.2^°^) were greater with the active brace than with no device (−2.3^°^ ± 1.7^°^ and −1.3^°^ ± 0.7^°^, respectively). The active brace (0.89 ± 0.07 s) was associated with a 17% shorter step time during incline walking compared to no devices (1.06 ± 0.09 s). It was also associated with a 62% longer step length during decline walking compared to no devices (0.27 ± 0.04 vs. 0.17 ± 0.03 m).

**Conclusion:**

In this case report, a pneumatic robotic knee wearable device improved quadriceps function and increased walking speed while freeing one arm to perform other tasks. A training program that optimizes function and comfort may improve device adoption as patients may need time to become acquainted with external torque delivery to the limb.

## 1. Introduction

Exoskeletons and robotic wearables can provide quadriceps muscle augmentation for patients who cannot get their quadriceps muscle completely repaired or have a torn or permanently weak knee extensor mechanism [1]. Patients with torn quadriceps or patellar tendons, patellar fractures, and anterior cruciate ligament tears may benefit from quadriceps augmentation if nonoperative treatment is pursued. Even postoperatively, these patients often have prolonged immobilization with weakened extensor mechanisms being a common complication [2, 3]. Early motion postoperatively has been associated with the promotion of healthy cartilage, improved tendon vascularity, and decreased time to regain full range of motion [2]. Patients with CNS disorders such as multiple sclerosis and L3 or L4 radiculopathies also exhibit weak extensor mechanisms and decreased mobility with improvements observed following rehabilitation with lower limb robotic devices [4, 5]. Chronic weakness of the knee extensor mechanism is also well documented in patients following total knee arthroplasty (TKA) and is associated with implant survival and patient outcomes [6]. Many TKA patients may have a considerable decrease in knee mobility postoperatively and may benefit from quadriceps augmentation [7].

Pneumatic exoskeletons are rigid, externally wearable devices intended to support joint function through torque assistance using pressurized gas to ease load bearing and/or improve strength [8]. Previous conventional metal‐based exoskeletons had limitations associated with the size and weight of the wearable device [9]. However, the lightweight pneumatic robotic wearable presented here weighs less than 6.8 kg (15 lbs) and was made possible by the use of air compression rather than traditional motors to induce torque. There have been limited studies on exoskeleton use for quadriceps augmentation. In this case report study, we aimed to determine how a lightweight pneumatic robotic wearable around the knee would affect gait and knee joint biomechanics.

## 2. Case Presentation

**Figure 1:**
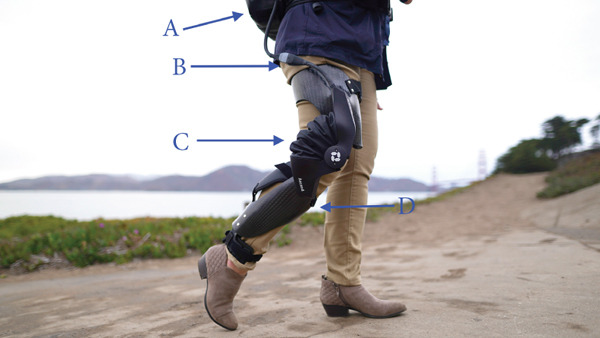
Diagram showing the pneumatic robotic knee wearable, Ascend, and its different components: (A) power pack, (B) remote, (C) pneumatic actuator, and (D) leg brace.

This case report on a lightweight pneumatic robotic wearable was performed at Stanford Hospitals and Clinics, Department of Orthopedic Surgery (Palo Alto, California) and the Motion Analysis & Sports Performance Laboratory at Lucile Packard Children′s Hospital at Stanford (Sunnyvale, California). The pneumatic robotic knee wearable, named Ascend, was produced in collaboration with Roam Robotics (San Francisco, California). The device consists of a leg brace which is affixed to the participant′s leg and a small power pack that contains the battery and power components (Figure 1). The device is designed to fit users who are between 1.5 m (5 ft) and 2.0 m (6 ft–6 in.) in height and between 63 kg (140 lbs) and 120 kg (265 lbs) in weight.

The complete device weighs approximately 6 kg, with the brace weighing 1 kg and the power pack weighing 5 kg. The brace consists of a series of straps attached to the user′s leg to keep the device′s hinge aligned with the user′s knee joint throughout normal operation. The power pack can be worn on the shoulder for mobile activities or placed on the ground near the user for relatively stationary maneuvers such as prolonged standing or sit‐to‐stand transitions. The power pack contains the battery, primary electronics board, and the mobile pneumatic power components required to power the system.

The wearable operates with a pneumatic power train and a novel pneumatic actuator made from high‐strength fabrics located at the knee joint. It detects user movement and intent (e.g., sitting, standing, walking, and stairs) using onboard sensors. Using this intent recognition, the orthosis can provide the appropriate amount of torque throughout all phases of gait, scaled according to user input through the sensors, supporting 120° range of motion around the knee joint. Through the range of power levels and methods of torque application, the device can produce 10%–50% of the torques generated biologically by healthy users in different activities of daily living. The user remains in control of the system throughout the operation and can adjust the desired assistance level or disable the system with an accessible remote. When disabled, a key feature of the pneumatic drivetrain is that the system operates like a passive brace and provides no impedance to normal motion.

The patient was a 68‐year‐old bull farmer who visited the Stanford Orthopaedic Arthritis Clinic due to ambulatory fatigue in his left knee following a chronic unrepaired transverse patellar fracture. He is 1.90 m tall (6 ft 3 in.) and weighs 109.8 kg (242 lbs) and does not have any significant past medical history. He was not a candidate for TKA with extensor mechanism repair given his lack of arthritis. An extensor mechanism reconstruction in the setting of a native knee would likely leave him with a stiff knee and limit his function further, so we sought the approval of a pneumatic robotic wearable and biomechanical assessment of the device. The protocol was approved by the Stanford University Institutional Review Board, and the patient provided informed consent.

A left‐sided pneumatic robotic wearable was provided to the patient, and he fitted the brace on himself under medical supervision. Velcro straps were used to secure the device, with two above the knee joint and two underneath the knee joint. The support setting of the device was set at Level 3 out of 5.

### 2.1. Gait Analysis

**Figure 2:**
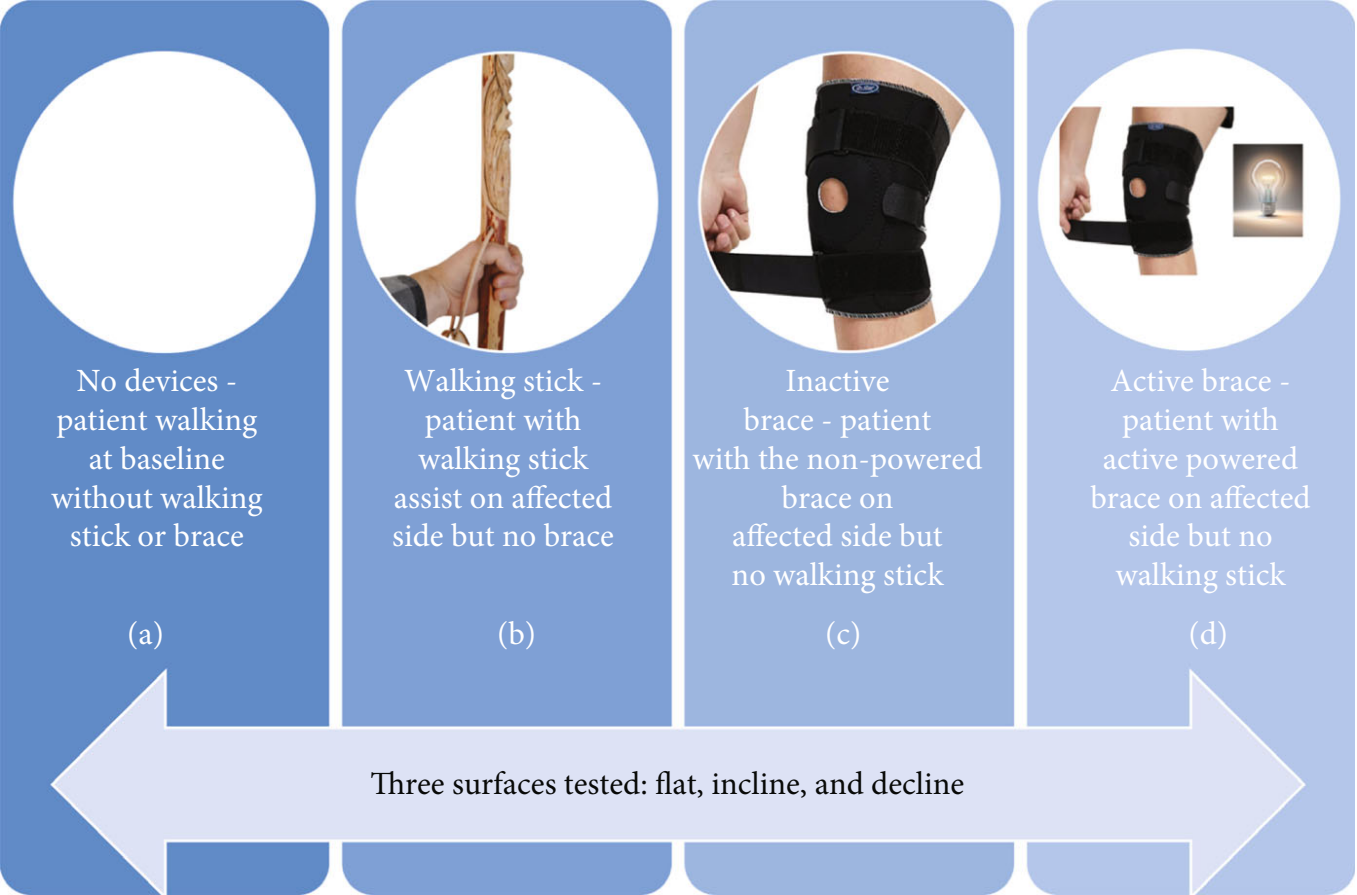
Experimental protocol demonstrating our four studied conditions: (a) the patient walking without an assistive device or a brace (no devices), (b) the patient walking with walking stick assist on the affected side but without a brace (walking stick), (c) the patient walking without stick assist but with a mechanical brace that is not activated on the affected side (inactive brace), and (d) the patient walking without stick assist but with active mechanical brace assist on the affected side (active brace).

All clinical measures that were recorded as part of the gait analysis had an experienced physical therapist present. In order to test the functional effectiveness of the pneumatic robotic wearable, gait analysis was performed under four conditions as seen in Figure 2: (A) the patient walking without an assistive device or brace (no devices), (B) the patient walking with a walking stick assist on the affected side but without a brace (walking stick), (C) the patient walking without stick assist but with an inactivated mechanical brace on the affected side (inactive brace), and (D) the patient walking without stick assist but with an active mechanical brace on the affected side (active brace). For each of the four conditions, walking was tested on three surfaces: flat, decline, and incline.

Flat walking was tested along an 8‐m path, and incline/decline walking included the addition of a 3‐m ramp. All testing was performed at the subject′s self‐selected speed. Kinematic (motion) and kinetic (force) data were recorded using a 22‐camera system (Vicon Motion Systems Ltd., Centennial, Colorado) with five integrated force plates (Bertec Corporation, Columbus, Ohio; Advanced Mechanical Technology Inc., Watertown, Massachusetts). Data was recorded in Vicon Nexus 2.9.1 where the motion data was captured at 120 Hz and force data was recorded at 1200 Hz.

To collect walking mechanics, 44 retroreflective markers were placed on the subject. Data collection started with a static calibration trial. After the static trial, four calibration markers located on the right and left medial epicondyle of the femur and medial malleoli were removed. The knee joint center was defined as the midpoint between the lateral and medial knee joint axes, and the ankle joint center was defined as the midpoint between the lateral and medial malleoli. In conditions where the brace was applied to the subject, some markers needed to be placed on the brace itself. This is a potential source of error, but adjustments were made to the knee joint width to ensure that the joint center was accurately located within the joint. Following subject calibration, the subject performed a minimum of three successful trials of flat walking, walking on an inclined surface, and walking on a declined surface.

All data was processed in Vicon Nexus 2.9.1. Motion data and force data were smoothed using a fourth‐order Butterworth filter using cut‐off frequencies of 12 and 50 Hz, respectively. To extract variable outputs, a custom written MATLAB script was utilized.

### 2.2. Statistical Analysis

Descriptive statistics from the patient′s gait analysis were summarized, with categorical data reported as frequencies and continuous data reported as mean and standard deviation (SD). Interknee comparisons were done for multiple gait parameters as well. Biomechanical variables extracted using a custom written MATLAB script included walking speed, step length, step width, peak hip and knee extensor moments, and sagittal and frontal plane kinematics of the hip and knee joints. A one‐way ANOVA was performed via IBM SPSS Version 27 (IBM, Armonk, New York) in order to analyze the difference in biomechanical variables between the four conditions. Flat, incline, and decline walking was analyzed separately. An alpha value of 0.05 was used to denote statistical significance.

## 3. Results

Our patient subjectively expressed satisfaction with the pneumatic robotic wearable and stated that he experienced a benefit over walking with his usual assist stick in his daily activities. Initial physical therapy assessment revealed modified independence in ambulation with 60° range of motion in the affected left knee and 120° range of motion in the healthy right knee.

### 3.1. Flat Walking

During flat walking, the active brace had the highest mean walking speed (0.41 ± 0.03 m/s) compared to no devices (0.28 ± 0.04 m/s), walking stick (0.39 ± 0.02 m/s), and inactive brace (0.39 ± 0.06 m/s, Table 1). Mean cadence for the active brace was 65.8 ± 6.5 steps/min compared to 58.4 ± 3.2 steps/min for the no device group. Mean step length for the active brace was 0.31 ± 0.02 m compared to 0.34 ± 0.01 m for the no device group. Post hoc analysis of walking speed showed that the active brace and inactive brace were associated with a 45% and 40% faster walking speed than the no device condition, respectively. The increase in walking speed for the active brace over no devices may be attributed to the 18% (0.19 m/s) decrease in step time created by the active support. Notably, the walking speed for the walking stick condition was similar to that of the active brace and was an improvement over walking with no devices. Analysis of knee flexion/extension angles, hip abduction/adduction angles, and ground reaction force data across the gait cycle is shown (Figure 3). Knee flexion at initial contact for the active brace (19.0^°^ ± 0.2^°^) and inactive brace (20.8^°^ ± 1.1^°^) was greater than no devices (−2.3^°^ ± 1.7^°^) and walking stick conditions (−0.2°). The inactive brace (20.8^°^ ± 0.9^°^) and active brace (19.0^°^ ± 0.2^°^) had greater peak knee flexion during stance than the no devices (−1.3^°^ ± 0.7^°^) and walking stick conditions (−0.5^°^ ± 0.4^°^).

**Table 1:** Comparison of gait parameters for left leg using a pneumatic robotic wearable with different conditions when walking on a flat surface.

**Gait parameters**	**No device**	**Walking stick**	**Inactive brace**	**Active brace**	**p** **value**	**Right leg**
Cadence (steps/min)	58.4 ± 3.2	64.5 ± 1.3	61.5 ± 3.7	65.8 ± 6.5	0.203	58.9 ± 4.97
Walking speed (m/s)	0.28 ± 0.04	0.39 ± 0.02	0.39 ± 0.06	0.41 ± 0.03	0.014 ^∗^	0.27 ± 0.04
Step length (m)	0.34 ± 0.01	0.35 ± 0.08	0.34 ± 0.04	0.31 ± 0.02	0.791	0.26 ± 0.05
Step time (s)	1.08 ± 0.10	0.98 ± 0.06	0.99 ± 0.05	0.89 ± 0.05	0.001 ^∗^	1.01 ± 0.18
Step width (m)	0.33 ± 0.02	0.30 ± 0.02	0.30 ± 0.02	0.32 ± 0.02	0.036 ^∗^	0.32 ± 0.02
Stride length (m)	0.60 ± 0.08	0.80 ± 0.07	0.72 ± 0.08	0.73 ± 0.03	N/A	0.56 ± 0.052

Abbreviation: N/A, not applicable.

^∗^Significance: *p* < 0.05.

Figure 3Walking on a flat surface. (a) The flexion and extension angles of the patient′s left knee under each of the four conditions: no devices (blue line), walking stick (orange line), inactive brace (gray line), and active brace (yellow line). (b) The abduction and adduction angles of the patient′s left hip under each condition. (c) Ground reaction force on the patient′s left leg under each condition.

**Figure:**
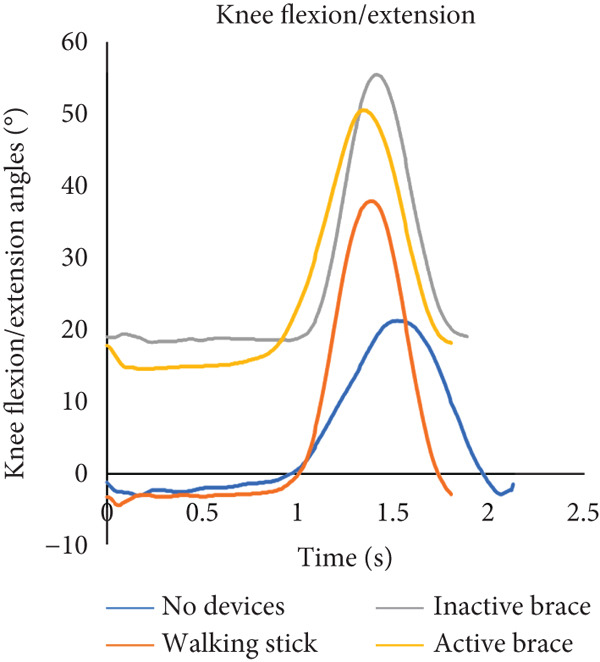
(a)

**Figure:**
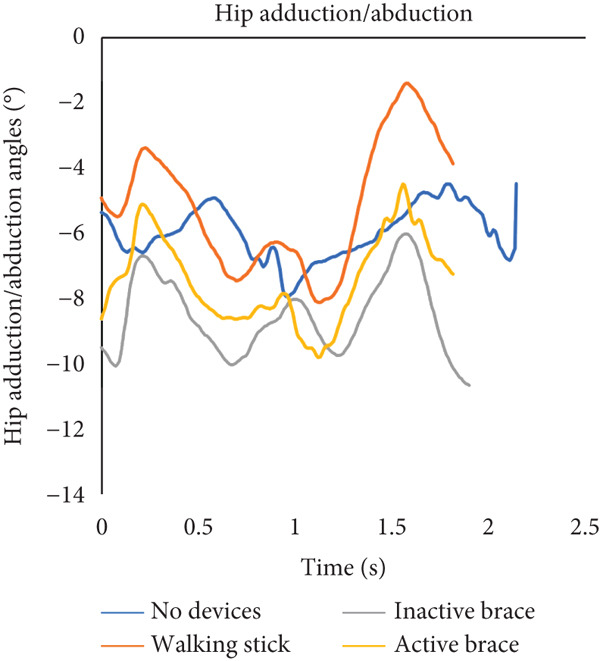
(b)

**Figure:**
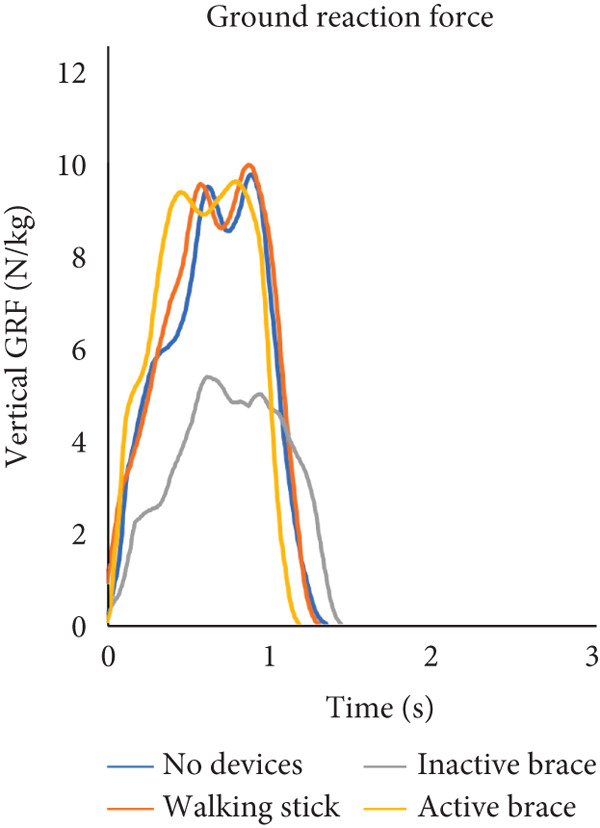
(c)

### 3.2. Incline Walking

During incline walking, the walking stick had the highest mean walking speed (0.40 ± 0.01 m/s) compared to no devices (0.31 ± 0.03 m/s), inactive brace (0.30 ± 0.07 m/s), and active brace (0.30 ± 0.13 m/s, Table 2). Mean step time was the lowest in the inactive brace (0.84 ± 0.05 s), followed by the active brace (0.89 ± 0.07 s) compared to no devices (1.06 ± 0.09 s) and walking stick (1.01 ± 0.01 s). In addition, the active brace (21.0^°^ ± 1.4^°^) and inactive brace (21.7^°^ ± 1.7^°^) had higher knee flexion at initial contact compared to the no devices (−0.6^°^ ± 0.3^°^) and walking stick conditions (−3.3^°^ ± 0.8^°^, Figure 4).

**Table 2:** Comparison of gait parameters for left leg using a pneumatic robotic wearable with different conditions when walking up an inclined surface.

**Gait parameters**	**No device**	**Walking stick**	**Inactive brace**	**Active brace**	**p** **value**	**Right leg**
Cadence (steps/min)	61.4 ± 4.0	63.9 ± 4.2	59.2 ± 10.6	59.5 ± 4.5	0.865	61.4 ± 4.46
Walking speed (m/s)	0.31 ± 0.03	0.40 ± 0.01	0.30 ± 0.07	0.30 ± 0.13	0.549	0.30 ± 0.03
Step length (m)	0.26 ± 0.02	0.36 ± 0.01	0.28 ± 0.06	0.27 ± 0.07	0.251	0.32 ± 0.03
Step time (s)	1.06 ± 0.09	1.01 ± 0.01	0.84 ± 0.05	0.89 ± 0.07	0.003 ^∗^	0.92 ± 0.07
Step width (m)	0.32 ± 0.02	0.27 ± 0.01	0.29 ± 0.01	0.28 ± 0.02	0.008 ^∗^	0.32 ± 0.02
Stride length (m)	0.58 ± 0.08	0.66 ± 0.27	0.59 ± 0.04	0.62 ± 0.06	N/A	0.58 ± 0.04

Abbreviation: N/A, not applicable.

^∗^Significance: *p* < 0.05.

Figure 4Walking up an inclined surface. (a) The flexion and extension angles of the patient′s left knee under each of the four conditions: no devices (blue line), walking stick (orange line), inactive brace (gray line), and active brace (yellow line). (b) The abduction and adduction angles of the patient′s left hip under each condition.

**Figure:**
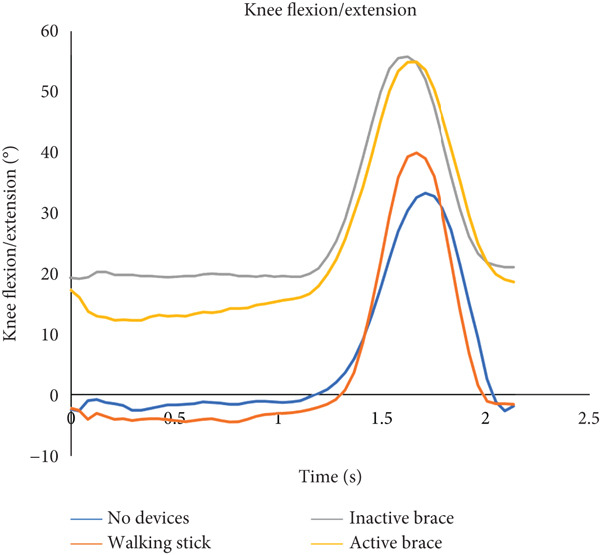
(a)

**Figure:**
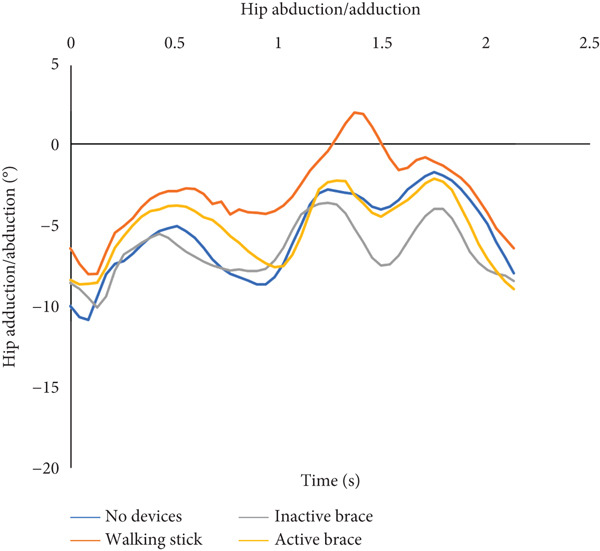
(b)

### 3.3. Decline Walking

During decline walking, the active brace had the highest mean walking speed (0.30 ± 0.05 m/s) compared to no devices (0.19 ± 0.09 m/s), walking stick (0.29 ± 0.01 m/s), and inactive brace (0.27 ± 0.01 m/s, Table 3). Mean step length for the active brace (0.27 ± 0.04 m) was the highest compared to no devices (0.17 ± 0.03 m), walking stick (0.25 ± 0.05 m), and inactive brace (0.22 ± 0.01 m). The active brace (18.6^°^ ± 2.1^°^) and inactive brace (19.8^°^ ± 1.1^°^) also had a higher knee flexion at initial contact compared to the no devices (−0.7^°^ ± 3.2^°^) and walking stick conditions (−1.4^°^ ± 1.1^°^, Figure 5).

**Table 3:** Comparison of gait parameters for left leg using a pneumatic robotic wearable with different conditions when walking down a declined surface.

**Gait parameters**	**No device**	**Walking stick**	**Inactive brace**	**Active brace**	**p** **value**	**Right leg**
Cadence (steps/min)	63.2 ± 6.7	66.6 ± 2.1	71.8 ± 2.3	69.0 ± 5.5	0.206	60.8 ± 11.5
Walking speed (m/s)	0.19 ± 0.09	0.29 ± 0.01	0.27 ± 0.01	0.30 ± 0.05	0.113	0.22 ± 0.08
Step length (m)	0.17 ± 0.03	0.25 ± 0.05	0.22 ± 0.01	0.27 ± 0.04	0.028 ^∗^	0.22 ± 0.04
Step time (s)	0.84 ± 0.08	0.86 ± 0.08	0.82 ± 0.08	0.80 ± 0.07	0.634	0.99 ± 0.16
Step width (m)	0.32 ± 0.03	0.28 ± 0.01	0.30 ± 0.01	0.31 ± 0.03	0.088	0.32 ± 0.03
Stride length (m)	0.43 ± 0.08	0.53 ± 0.09	0.50 ± 0.04	0.51 ± 0.05	N/A	0.42 ± 0.08

Abbreviation: N/A, not applicable.

^∗^Significance: *p* < 0.05.

Figure 5Walking down a declined surface. (a) The flexion and extension angles of the patient′s left knee under each of the four conditions: no devices (blue line), walking stick (orange line), inactive brace (gray line), and active brace (yellow line). (b) The abduction and adduction angles of the patient′s left hip under each condition.

**Figure:**
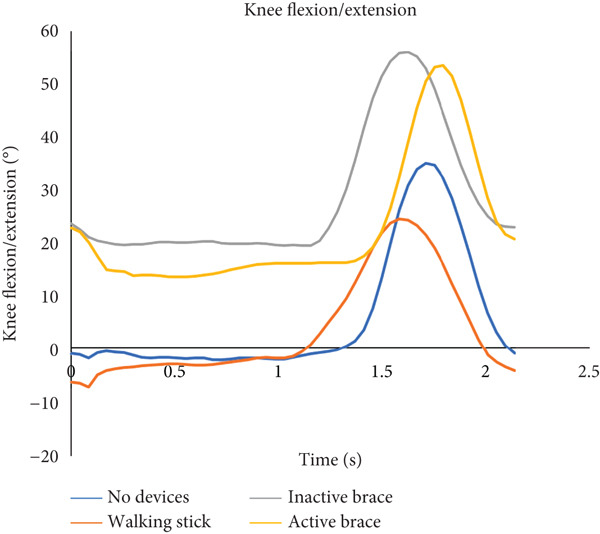
(a)

**Figure:**
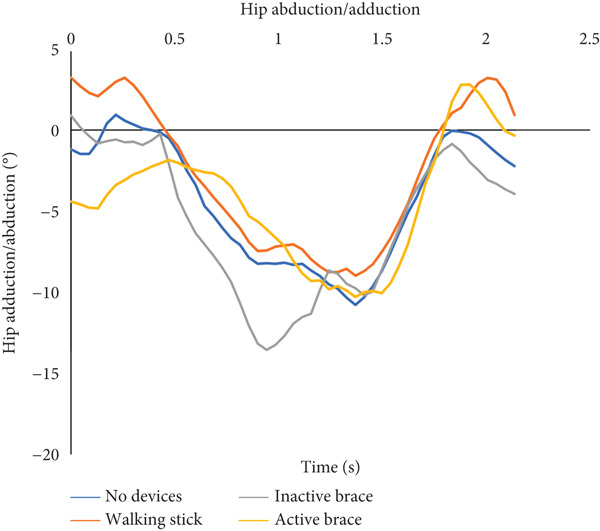
(b)

## 4. Discussion

In this case report, we demonstrated that the use of a pneumatic robotic wearable was associated with a perceived benefit by the patient and an increased walking speed. The active brace provided quadriceps and lower limb muscle augmentation which decreased our patient′s step time by 18% and increased his walking speed by 45% on a flat surface compared to his baseline. The active brace serves as a more efficient assist than the patient′s usual walking stick, which was like walking with no devices on many biomechanical variables including peak knee flexion. On the other hand, the active brace improved peak knee flexion to 19° from knee hyperextension without the brace. Although walking speed was similar between the active brace and walking assist (0.41 vs. 0.39 m/s), the robotic device did free up one of the patient′s arms for other tasks without compromising on other gait parameters. Having an extra hand may drastically improve the ease of daily activities and overall quality of life. Biomechanically, the robotic wearable may perform just as good, if not better, than the walking assist which may contribute to the patient′s perceived benefit of the lightweight pneumatic robotic wearable device.

Patients undergoing TKA comprise a large patient population that may benefit from quadriceps muscle augmentation given the significant need for assistance and rehabilitation postoperatively [10]. For example, weakened knee extensor mechanisms postoperatively directly contribute to a functional decline in lower limb strength and gait impairment [10, 11]. Several studies have documented that TKA patients exhibit reduced peak knee mobility compared to healthy controls [12, 13]. In addition, TKA patients commonly exhibit step length asymmetry, slower walking speed, and altered biomechanics [14, 15]. Gait impairment 1–2 years after TKA is even suggestive of incomplete recovery of the knee joint [16]. Up to 20% of TKA patients express some level of dissatisfaction due to knee pain or limited knee mobility postoperatively, but lightweight pneumatic robotic wearables may serve as a potent tool for recovery and muscle augmentation [17, 18]. This may be especially relevant as the demand for primary TKAs is expected to grow to 3.5 million procedures annually by 2030 in the face of our country′s aging population and obesity epidemic [19].

The reductions in metabolic cost associated with exoskeletons when comparing their active support‐delivering state and inactive state are well documented in the literature [20–22]. Although we did not measure metabolic cost, we did notice improvements in step time and walking speed when comparing the active brace to the inactive brace, which are likely congruent with a reduced metabolic cost due to muscle augmentation. There were several key areas where differences were not observed between the active and inactive brace. For example, both brace conditions had similar increases in knee flexion at heel strike compared to nonbrace equipped conditions, which suggests that some of the differences in gait and biomechanical variables associated with the pneumatic robotic wearable may be related to the presence of the device itself and not necessarily its activation.

Of the studies that focused on metabolic cost, only one assessed incline walking and none assessed decline walking [22]. Given that knee extensor moments are increased by up to 500% during incline walking, the 17% shorter step time we found with the robotic wearable may be associated with the device′s augmentation of quadriceps muscle knee extension [22]. The 62% increase in step lengths and faster walking speed associated with only the active brace during decline walking suggest that the brace actively performed negative work. It may be acting as a shock absorber for force that would otherwise be loaded onto the surrounding muscles and joint, which is consistent with its effect in reducing metabolic cost. The improvements in gait associated with exoskeletons during decline walking are not well documented, but given that the amount of incline walking and decline walking performed in daily activities should be similar for most patients, this underscores the need for additional research.

We note that the ground reaction force data presented in Figure 3 may appear counterintuitive. The inactive brace had a lower peak ground reaction force (5.4 N/kg) compared to the other walking conditions (range: 9.7–10.1 N/kg) even though one would expect similar and increased ground reaction forces with the inactive brace and active brace given the additional weight of the brace. It is possible that there is offloading of the affected limb with the inactive brace with the contralateral limb, although we did not note any large differences in the contralateral ground reaction forces in the inactive brace (9.3 N/kg) compared to the other groups (range 9.5–9.6 N/kg). This may be partially related to the case report nature of this study and should be recognized as a limitation.

Exoskeletons and robotic devices can come with power or support settings to determine the amount of active support the user desires. For this study, the support of the robotic wearable was set at Level 3 (out of 5) which our patient was pleased with. Notably, he had initially tried Level 4 but stated that it was “uncomfortable.” However, it is possible that Level 4 may be the “right” setting and that the uncomfortable feeling our patient is experiencing stems from the high levels of force and torque being generated through his knee. Coaching may be necessary to optimize the trade‐off between comfort and function to gain the maximal benefit from the device. Other studies using exoskeletons for different applications have included long training periods, up to 20 1‐h sessions, with an experienced technician, which suggests that training can play a large role in helping the patient fully maximize the brace [23, 24]. Battery life and charging are additional areas where a patient will directly interact with the device and can impact the patient experience. This version of the active brace has approximately 90 min of run time across all support settings and takes 90–120 min to fully recharge. Although it may require charging during the day, the trade‐off between battery life and device weight must be considered. Given that patients requiring quadriceps augmentation may spend only a limited amount of time in continuous movement requiring active brace support, the extra weight from additional batteries may prove more cumbersome than beneficial.

Our study was not without limitations. Although we completed multiple trials for each brace condition and walking condition, we only examined one patient′s interaction with the brace and may have been underpowered to detect statistically significant differences. In addition, we only assessed our user′s interaction with the device at one time point. We also did not acquire formal or standardized patient‐reported outcome measures due to the primarily biomechanical nature of this study. It may be beneficial for future studies to examine how the pneumatic robotic wearable impacts gait in both a larger cohort of patients with weak extensor mechanisms and over a longer follow‐up period with formal patient‐reported outcomes to determine when it has its most beneficial effect. Although technical details of the device are helpful in evaluating biomechanical data, we are unable to provide additional information besides what is already discussed on torque output, battery performance, safety features, etc. due to the proprietary nature of this work. Next steps also include examining battery usage between different tasks and whether training can allow patients to make the most of higher support levels.

In conclusion, in this case report, we demonstrated that a lightweight pneumatic robotic wearable contributed to significant improvements in gait through a shorter step time and increased walking speed, which led to a perceived benefit by the patient. It was a more efficient ambulatory assist that also freed one of the patient′s arms, which would otherwise be holding a walking stick, to perform other tasks. Although future studies are needed, pneumatic robotic devices may be applicable for patients requiring quadriceps muscle augmentation such as TKA patients postoperatively due to its effects on improving joint biomechanics, function, and overall quality of life.

## Conflicts of Interest

D.F.A. reports grants from NIH‐NCATS and OREF; consultancy for J&J MedTech, Exactech, Medacta, Corin, Heraeus, and Bone Support; expert testimony for the Expert Institute; patents owned by Knimble Designs, Zooly Labs, Arthology Consulting, PlantarTech, and Stanford University; royalties from Corin, Exactech, and Medacta; and stock/stock options in *n*Sight Surgical, Knimble Designs, Zooly Labs, QT Ultrasound, Recoup Fitness, and Well Beam all unrelated to the submitted work. All other authors have no conflicts to report.

## Author Contributions

D.F.A. conceived the idea. S.L., P.A., J.J.M., and S.C. contributed to data collection. S.L., and J.J.L. performed data analysis. J.J.L. and D.F.A. prepared the manuscript.

## Funding

All authors did not have any relevant funding related to the study design, data collection, analysis, interpretation of data, writing the report, or the decision to submit the findings for publication.

## Data Availability

The data that supports the findings of this study are available from the corresponding author upon reasonable request.
